# A Review on Conventional and Advanced Methods for Nanotoxicology Evaluation of Engineered Nanomaterials

**DOI:** 10.3390/molecules26216536

**Published:** 2021-10-29

**Authors:** Anny Leudjo Taka, Charlotte Mungho Tata, Michael John Klink, Xavier Yangkou Mbianda, Fanyana Moses Mtunzi, Eliazer Bobby Naidoo

**Affiliations:** 1 Department of Chemistry/Biotechnology, Vaal University of Technology, Vanderbijlpark 1900, South Africa; fanyana@vut.ac.za (F.M.M.); bobbyvut@gmail.com (E.B.N.); 2 Institute of Chemical & Biotechnology, Vaal University of Technology, Southern Gauteng Science and Technology Park, Sebokeng 1983, South Africa; 3 Department of Chemical Sciences, University of Johannesburg, Doornfontein, Johannesburg 2028, South Africa; ttcharlym@yahoo.com (C.M.T.); mbianday@uj.ac.za (X.Y.M.); 4 Department of Biochemistry, University of Bamenda, Bambili 00237, Cameroon

**Keywords:** nanotoxicology, nanocomposite, nanomaterial, apoptosis, oxidative stress

## Abstract

Nanotechnology can be defined as the field of science and technology that studies material at nanoscale (1–100 nm). These nanomaterials, especially carbon nanostructure-based composites and biopolymer-based nanocomposites, exhibit excellent chemical, physical, mechanical, electrical, and many other properties beneficial for their application in many consumer products (e.g., industrial, food, pharmaceutical, and medical). The current literature reports that the increased exposure of humans to nanomaterials could toxicologically affect their environment. Hence, this paper aims to present a review on the possible nanotoxicology assays that can be used to evaluate the toxicity of engineered nanomaterials. The different ways humans are exposed to nanomaterials are discussed, and the recent toxicity evaluation approaches of these nanomaterials are critically assessed.

## 1. Introduction

Nanomaterials (e.g., metal nanoparticles, carbon nanostructures) and their hybrid nanocomposites (with polymers) exhibit exceptional physicochemical, mechanical, thermal, optical, and electrical properties due to their quantum effects and large surface to volume ratio. They are considered as a promising approach to resolve several issues in various fields, such as medical, textile, food, drug delivery, electronics, and environmental cleanup [1]. However, the increased use of nanomaterials and their hybrid nanocomposites in many consumer products has led to potential human exposure to these nanostructures through inhalation, ingestion, and skin contact. This has resulted in adverse side effects due to nanotoxicity, especially on the central nervous system, circulatory system, gastrointestinal tract, and respiratory system. For example, carbon-based nanomaterial products dispensed via injection have been shown to enter the circulatory system, causing secondary complications [2].

Moreover, the toxicity evaluation of these nanomaterials (and their hybrid composites), which is crucial for their safe use in consumer products, remains challenging. For instance, there are limited reports presenting a detailed understanding of carbon nanostructures’ toxicity and their composites. In these reports, the in vitro and in vivo studies on toxicological effects are not explicit enough, and the data are hard to interpret [1,3].

Therefore, more research on the nanotoxicity assessment of nanomaterials and their hybrid composites is required. In this article, the different approaches (conventional and advanced) used to assess the toxicity of nanomaterials are reviewed. The possible mechanisms of nanomaterial toxicity and the factors affecting toxicity are also discussed.

## 2. Nanotoxicology and the Different Factors Influencing the Toxicity of Nanomaterials

Nanotoxicology is a branch of toxicology that evaluates the adverse effects of nanomaterials (NMs) on human health and the environment to better comprehend and assess the health risks attached to their use [2,4]. The factors affecting the toxicity of NMs include their physicochemical properties, such as particle size, particle aggregation, chemical composition, surface area, shape, crystallinity, structure, surface functional groups/charge, surface coating, and reactivity [2,3]. These physicochemical properties result from the preparation methods used.

In particular, the surface area and shape of NMs affect the cell uptake mechanism. As a result, the NMs’ cytotoxicity depends on the shape of the particle. The literature also reveals that the nanoparticle dimension and shape determine its toxicity. Particle size and size distribution also affect cell uptake, the endocytosis process, the intracellular fate of NMs, and the cytotoxicity response (e.g., NMs with smaller sizes have been reported to show a higher cytotoxicity response). In addition, surface coating of NMs has been shown to act as an interface between the cell and the NMs by affecting the intrinsic interactions, cytotoxicity, and cell uptake. For example, NMs with positive charges have been demonstrated to produce more toxicity effects than the NMs with negative and neutral charges [5,6].

Furthermore, the surface nanostructures promote the sorption of ions and molecules, which affect cellular responses and cause toxicity [2]. Additionally, the genetic composition of the organism exposed to nanoparticles (NPs), the ability of the NPs to be stable in biological systems, the longevity of NP–cell interaction, dose, frequency, duration, and route of exposure are also some factors influencing toxicity [7,8,9,10].

## 3. Possible Mechanism of Nanomaterial Toxicity

**Figure 1:**
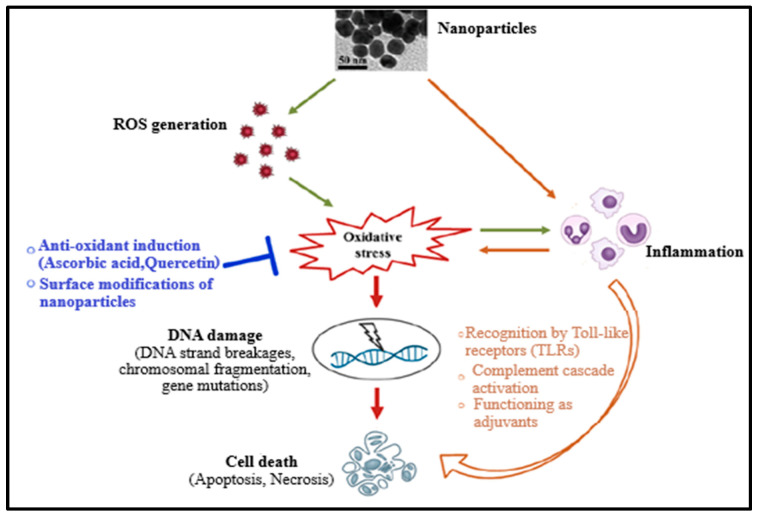
Mechanism of the nanomaterials’ toxicity [2].

Up to date, the mechanism of nanomaterial toxicity has not yet been clearly defined due to the inconsistency of nanotoxicity data obtained. However, based on the results already published, it has been reported that NMs can cause reactive oxygen species (ROS) production either directly or indirectly during toxicity or cytotoxicity, leading to oxidative stress (Figure 1). This could be due to the existence of pro-oxidant functional groups on their reactive surfaces or NM–cell interactions [2,11,12,13]. Oxidative stress can cause damage of DNA (which includes induction of gene mutations, DNA strand breakages, and chromosomal fragmentation) and subsequently apoptosis. NMs (or nanoparticles) have also been reported to induce inflammation, resulting in toxicity and promoting cell death by inducing toxic by-products like complement proteins and ROS or receptor-induced necrosis/apoptosis (Figure 1) [2,10,12,13,14]. Furthermore, oxidative stress may provoke the release of pro-inflammatory mediators through phosphoinositide 3-kinase (PI3-K) pathways, mitogen-activated protein kinase (MAPK), and nuclear factor-κB (NF-κB), indicating a mutual correlation between oxidative stress and inflammation. Therefore, the damage caused by nanoparticles to the cell is cytotoxic and genotoxic [2,10,12].

**Figure 2:**
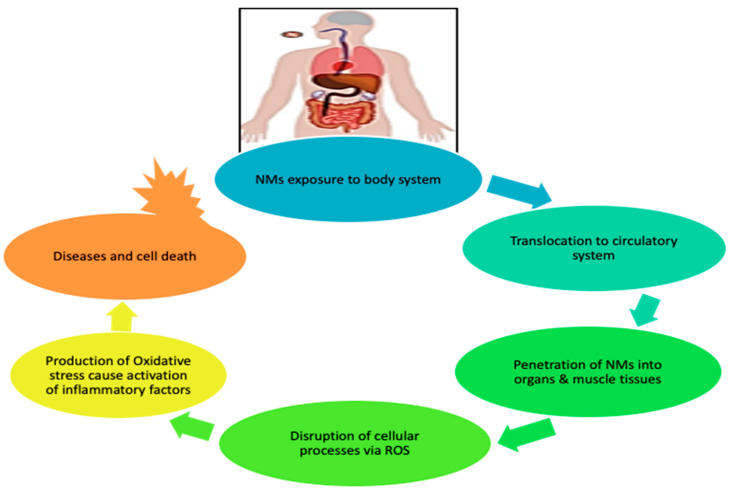
Effect of engineered nanomaterials (ENMs) upon exposure to human body.

Figure 2 further illustrates the pathway taken by NMs on exposure to the human body. The NMs are capable of penetrating the pulmonary system, gastrointestinal tract, and skin barriers, and then translocating to the circulatory system, where they are distributed to muscle tissues and organs (heart, brain, lung, kidney, liver); they disrupt cellular processes via ROS production, leading to oxidative stress. These processes later cause activation of inflammation, triggering the disease and cell death [5].

## 4. The Different Approaches to Evaluate the Toxicity of Nanomaterials

### 4.1. Conventional Methods

#### 4.1.1. In Vitro Methods

Conventional nanotoxicological evaluations are predominantly through in vitro cellular approaches because they are not costly and are less time-consuming than in vivo animal tests [15]. The parameters usually investigated in in vitro toxicity studies are oxidative stress, inflammatory changes, DNA damage/mutation, apoptosis, and cytotoxicity (or necrosis). Various experiments can be performed to evaluate these parameters [3,16,17,18]. Oxidative stress is determined by conducting tests, such as 2,7-dichlorofluorescein (DCFH) assay, electron paramagnetic resonance (EPR), and lipid peroxidation. Excess ROS production in the cytosol due to oxidative stress can be measured using a fluorophore membrane, 2,7-dichlorofluorescein diacetate (DCFH-DA), which is permeable and nonpolar. When the nonfluorescent DCFH-DA is taken up by cells, the fluorophore is enzymatically hydrolyzed by cytosolic esterases into its nonfluorescent polar analog dichlorofluorescein (DCFH), which is trapped within the cytosol. ROS then oxidizes the trapped DCFH into highly fluorescent dichlorofluorescein (DCF), which can be monitored using fluorescence microscopy or flow cytometry [16,19].

On the other hand, the electron paramagnetic resonance (EPR) test involves the use of 4-hydroxy-2,2,6,6-tetramethylpiperidine-1-oxyl (a radical-consuming spin probe) or 5,5-dimethyl-pyrrolidine N-oxide, DMPO (spin-trapping agent) for superoxide (O^2−^) or hydroxide (HO^−^) radicals, which are added into the NP or culture solution, for a specific time. After, the entire supernatant is vortexed, concentrated, and analyzed on an EPR spectrometer [20]. The lipid peroxidation test measures two main secondary peroxidation products: 4-hydroxyl-2-nonenal (4-HNE) and malondialdehyde (MDA), which are released from oxidative degradation of cell membranes. The reaction of MDA with thiobarbituric acid (TBA) forms an MDA-TBA adduct that is detected fluorescently or spectrophotometrically. Further, 4-HNE can be detected using HNE-protein adduct ELISA assays because of its high reactivity with primary amines to produce thiol (or amino compounds) and Schiff bases to obtain Michael adducts. In samples comprising 4-HNE and MDA, the reaction of TBA with both MDA and 4-HNE is highly promoted due to the nonspecific nature of the assay. However, in a reaction medium containing hydrochloric acid, TBA can be substituted with 1-methyl-2-phenylindole, which favors MDA adduct yields over 4-HNE [16,21].

Another parameter that can be measured via in vitro testing is inflammatory changes. The markers of inflammation can be detected through enzyme-linked immunosorbent assay (ELISA). These markers include numerous families of cytokines, such as monocyte chemotactic protein-1 (MCP-1) and interleukin 8 or 6 (IL-8 or IL-6), which are activated by the immune system against foreign agents like NPs. Cytokines expressed by proinflammatory and anti-inflammatory macrophages promote T-helper type 2 and 1 (Th2 and Th1) cell responses, respectively, whose activities lead to the release of interleukin-10 (IL-10) and tumor necrosis factor-alpha (TNF-α) within the extracellular medium, where their intensities can be estimated by ELISA [22,23].

Furthermore, cytotoxicity or necrosis assay can also be evaluated through in vitro assays. Cytotoxicity assays involve tests, such as Tetrazolium salts assay, Alamar blue assay, clonogenic assays, Trypan blue assay, and lactate dehydrogenase (LDH). Tetrazolium salts assay deals with the measurement of mitochondrial function of viable cells compared to the untreated cells (control). The cells are exposed to NPs for different times before the addition of reduced tetrazolium salts assay (e.g., XTT, MTT, MTS, or WST) and incubated at 37 °C for 2–4 h [24,25,26]. The viable cells with vital respiratory mitochondrial activity biodegrade MTT via mitochondrial succinic dehydrogenases into an insoluble purple formazan product, which can be dissolved afterward using DMSO. On the other hand, MTS or XTT are metabolized to a water-soluble formazan product, eliminating the use of DMSO for the solubilization step. Quantitation is then done using a visible light spectrophotometer. In addition, among these reduced tetrazolium salt assays, MTS is the most efficient since it produces accurate and more sensitive absorbance values, which quickly confirm positive results. WST is the least recommended because it cannot penetrate the cells requiring an extracellularly reduced plasma membrane [24,25,26]. For example, Muktha and co-workers reported on the use of the MTT assay to evaluate the cytotoxicity of carbon dots on MCF-7 and HepG2 cancer cell lines. They demonstrated that carbon dots prepared from pomegranate peels (P-C dots) and watermelon peels (W-C dots) both have strong anticancer effects against MCF-7 cell lines, while for HepG 2 cell lines, the cytotoxicity effects of the W-C dots and P-C dots were equal [27].

In the Alamar Blue assay, the active ingredient is a non-toxic water-soluble resazurin blue dye that is cell permeable and nonfluorescent. When the cells are treated with the blue resazurin dye, the viable cells degrade resazurin to resorufin by forming a fluorescent bright red color, whereas the presence of dead cells is indicated if the resazurin dye remains blue. This produces quantitative data on the cytotoxicity and viability [28]. Besides using the Alamar blue assay, the clonogenic assay can also be employed to evaluate the increase or decrease in cell viability as well as cell reproduction over some time. Cells are either pre-exposed to NMs before plating or treated with NMs following plating. After plating at a very low concentration, these cells can regenerate until colonies are observed. From a single cell plate, it is believed that a colony can be developed, and the obtained colonies are counted by visual inspection [29,30]. In contrast, the neutral red assay is based on viable cells’ ability to bind a supravital weak cationic neutral red dye that easily enters cell membranes through non-ionic diffusion and accumulates in lysosomes. Lysosomal defect and other variations become progressively unchangeable. Cytotoxicity is expressed as a concentration-dependent reduction of neutral red adsorption after exposure to chemicals, therefore giving a sensitive integrated signal of growth inhibition and cell integrity [31].

Additionally, Trypan blue and lactate dehydrogenase assays can also be considered for the investigation of cytotoxicity. In the Trypan blue assay, trypsinized cells are stained with diazo trypan blue dye, which is adsorbed only by dead cells [32]. Alternatively, the lactate dehydrogenase (LDH) assay measures the integrity of the cell membrane based on the oxidation of iodonitrotetrazolium (INT) (yellow tetrazolium) salt to a red formazan. As large amounts of LDH are discharged from the cytosol upon cellular apoptosis or necrosis, it catalyzes the conversion of pyruvate to lactate and the complimentary reaction of NADH with tetrazolium salts permits spectroscopic monitoring of LDH enzymatic activity using known initial concentrations of lactate and NAD [33]. Uboldi and co-workers examined the cytotoxicity of gold NPs (Au NPs) using the LDH assay in human alveolar type-II (ATII)-like cell lines A549 and NCIH441. They reported that the viability of NCIH441 and ATII-like cell lines A549 were affected by sodium citrate residues on Au NPs. The excess of sodium citrate on the Au NPs surface reduced the viability of the cell lines, affected cellular proliferation, and increased the release of LDH respectively by the KI-67 and LDH release assay [34]. Moreover, Table 1 describes other in vitro assays that can be used to assess the nanotoxicity.

Despite the fact that in vitro experiments are rapid, inexpensive, and reproducible, they also have some limitations, such a as lack of NM’s secondary interferences. Moreover, the results generated by in vitro nanotoxicity testing are quite conflicting. These inconsistencies could be due to NP–dye interaction or NP adsorption of the dye in tests where staining is required. However, despite the inconsistencies, in vitro testing still provides a primary direction in eventual toxicity studies and indicates the complexity involved in NPs-mediated toxicity [44]. To limit error, more than one type of in vitro experiment is usually required. In addition, there is little correlation between in vitro and in vivo studies. This is partially due to the inability of in vitro studies to mimic the complex systems and homeostasis maintained by clearance organs, such as the kidney and liver [25,45].

#### 4.1.2. In Vivo Methods

In vivo methods of evaluating nanotoxicity mainly involve the use of experimental animals, some of which include zebrafish (*Danio rerio*), *Caenorhabditis elegans*, Drosophila melanogaster, mice, rats, and rabbits [46]. The working principle of this method is as follow: orally administered NMs are captured by the gastrointestinal tract and translocated by blood to other organs, such as the liver, spleen, kidney, heart, lungs, and brain [3]. Other routes of exposure include inhalation, transdermal delivery, and injection. In vivo evaluation of nanotoxicity may be classified into two main categories: one involves inflammation infiltration, apoptosis, and tissue structure changes in the main organs (heart, brain, lung, spleen, and kidney), while the other targets specific systems that are subject to concentrated NMs, e.g., Kupffer cells of the liver, hepatic sinusoid, and renal filtration membrane [47]. Small rodents (e.g., mice, rats), zebrafish, Caenorhabditis elegans (*C. elegans*), and Drosophila melanogaster are the most widely used animal models to evaluate NMs’ nanotoxicity by the in vivo method [46].

Zebrafish are mostly preferred as animal models for the evaluation of nanotoxicity because they are small in size, highly reproducible, develop quickly with transparent embryos, and are compliant with genetic and chemical screens. Their cardiovascular, nervous, and digestive systems; physiological and immunogenic responses; anatomical coherence like blood–brain barrier; and endothelial cells are similar to that of mammals (or humans) [48]. Moreover, both zebrafish and humans have highly conserved signaling pathways with a high genomic homology level (approximatively 75% similarity), making it a feasible animal model for analytical studies [49]. In order to assess the toxicity in zebrafish, one or more of the following procedures can be conducted: evaluation of hatching, in vitro/in vivo imaging, skin and endocrine systems, behavioral analysis, transgenic zebrafish as live biosensor, mortality assessment, disruption of gills, reproductive toxicity, neurotoxicity, genotoxicity, immunotoxicity, and observing developmental malformation of organs and embryos [49,50,51,52,53]. Moreover, the results obtained from zebrafish are helpful for an efficient and rapid understanding of molecular and cellular mechanisms. For example, the nanotoxicity of ZnO nanoparticles (NPs) was evaluated by Choi and co-workers using the zebrafish model. They demonstrated that ZnO NPs induced the malformation of phenotypes, increased the mortality rate of zebrafish embryos, and affected the expression of inflammatory and immune response genes [54]. In addition, Duan and colleagues also demonstrated that silica NPs affect the neutrophils of zebrafish by causing an inflammatory response and destroying vascular endothelial cells [55].

*C. elegans* (worms) are small nematodes employed to evaluate the nanotoxicity of NPs through an in vivo system. The interaction of *C. elegans* with NPs can be used to determine nanotoxicity after acute, prolonged, and chronic exposure [56,57]. For instance, Kim et al. [58] used a microfluidic chip in which *C. elegans* was incorporated for in situ and in vivo Ag NP nanotoxicity evaluation. The working principle was as follows: in the absence or presence of Ag NPs, *C. elegans* were cultured in microfluidic chambers, then transferred to wedge-shaped channels, where they were immobilized to allow the assessment of factors, such as fluorescence emission (from the reporter gene), moving distance, and length, of *C. elegans* after exposure. From the results obtained, Kim and co-workers demonstrated that Ag NPs affect the length of *C. elegans*. They were able to detect a decrease in the body length on the chip’s channel, which in turn favors an increase in the worm’s (C. elegans) moving distance. The emission of the green fluorescence was also noted upon the uptake of Ag NPs. Hence, Kim et al. proved that *C. elegans*-on-a-chip has great potential to be made specific to NPs for a rapid detection system of nanotoxicity [58].

Drosophila melanogaster (*D. melanogaster*) are flies that are widely used to examine nanotoxicity in vivo. The use of the Drosophila model for in vivo studies has shown lower ethical and practical obstacles due to their low operational cost, ease of maintenance, relatively short life cycle, and reduced genetic redundancy [46,59]. For example, the nanotoxicity of Ag NPs induced the formation of reactive oxygen species, activated cytotoxic pathways, shortened the life span, and reduced the stress resistance capacity of *D. melanogaster* [60].

Small rodents (mice, rats, rabbits) have a close similarity to humans and are more convenient and affordable to maintain than larger animals (e.g., pigs, which are genetically very similar to humans). Small rodents can be used to examine various NP exposure routes, such as gavage, intra-tracheal installation, ingestion, intraperitoneal injections, intravenous injections, or inhalation. After exposing the small rodents to NPs for a period of time, blood and organ samples can be harvested and tested for hematological, biochemical, and histopathological changes. The bioaccumulation and/or distribution of NPs in vital organs/bodies can also be evaluated [53,61]. Additionally, other organisms used for in vivo nanotoxicity tests are presented in Table 2.

In summary, in vitro and in vivo methods are usually considered for the assessment of NM toxicity. Among all the assays, techniques, and organisms described above to evaluate the nanotoxicity of NMs, the LDH and MTT assays are mostly used in the in vitro assessment of NMs, whereas organisms, such as zebrafish, *C. elegans*, *D. melanogaster*, and small rodents (mice and rats), are commonly used in in vivo study. Moreover, these two methods also have their disadvantages. For instance, the disadvantages of in vitro assays include the lack of NM secondary interferences and unknown physiological pathways while the limitations of the in vivo approach are its inability to predict human biological responses, ethical issues, and the extended time required for the evaluation [6]. Besides this, there are drawbacks to establishing practical in vitro models analogous to in vivo testing models. For this reason, the literature has recommended that a series of tests, such as oxidative stress, cell viability, inflammatory response, genotoxicity, immunotoxicity, cell uptake, and transport, should be conducted to evaluate the nanotoxicity [12]. This approach helps to determine and establish different mechanisms for toxicity endpoints. In addition, animal models, such as zebrafish, Caenorhabditis elegans, and Drosophila melanogaster, should be considered as reliable models for in vivo nanotoxicity studies because of their cost efficiency, and they provide a reliable system for high-throughput screening processes necessary to construct structure–function relationships that favor modeling by the appropriate combination of in vivo and in vitro testing [26,46].

### 4.2. Advanced Approaches to Assess Nanotoxicity

The advanced approaches are techniques ranging from the use of cells, tissues, or organs-on-chips and tissue slices that greatly mimic in vivo environments, and ex vivo/in vivo methods and high-throughput methods, involving the use of advanced instruments, can also be employed to investigate nanotoxicity [12,15]. These advanced techniques are described in the following subsections.

#### 4.2.1. Atomic Force Microscopy (AFM)

**Figure 3:**
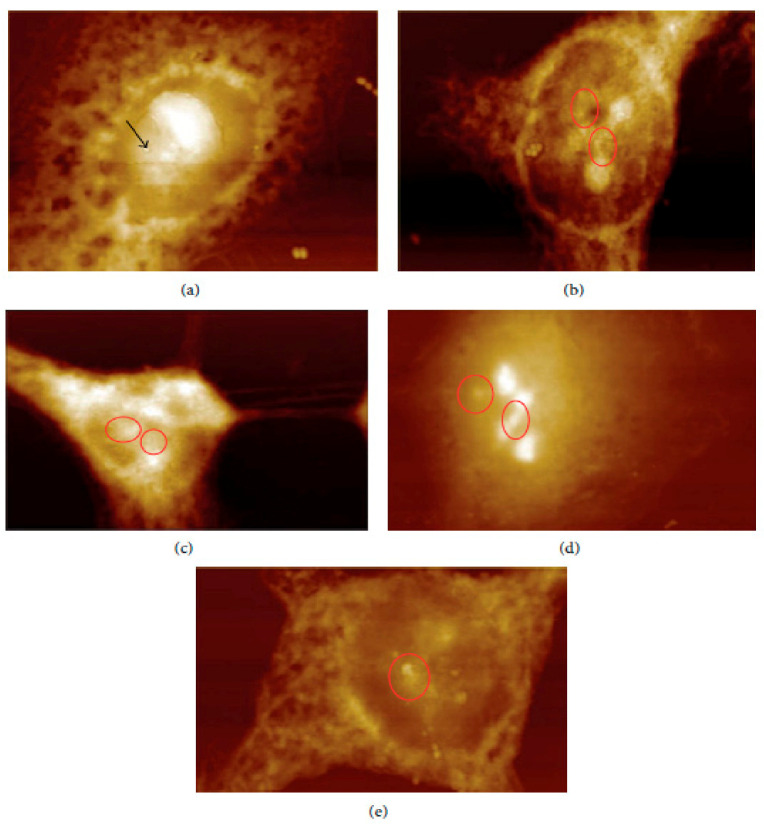
AFM images of 3-nm gold NPs into Hep-2 cells after different exposure times: (**a**) 1, (**b**) 2, (**c**) 4, (**d**) 12, and (**e**) 24 h [65].

**Figure 4:**
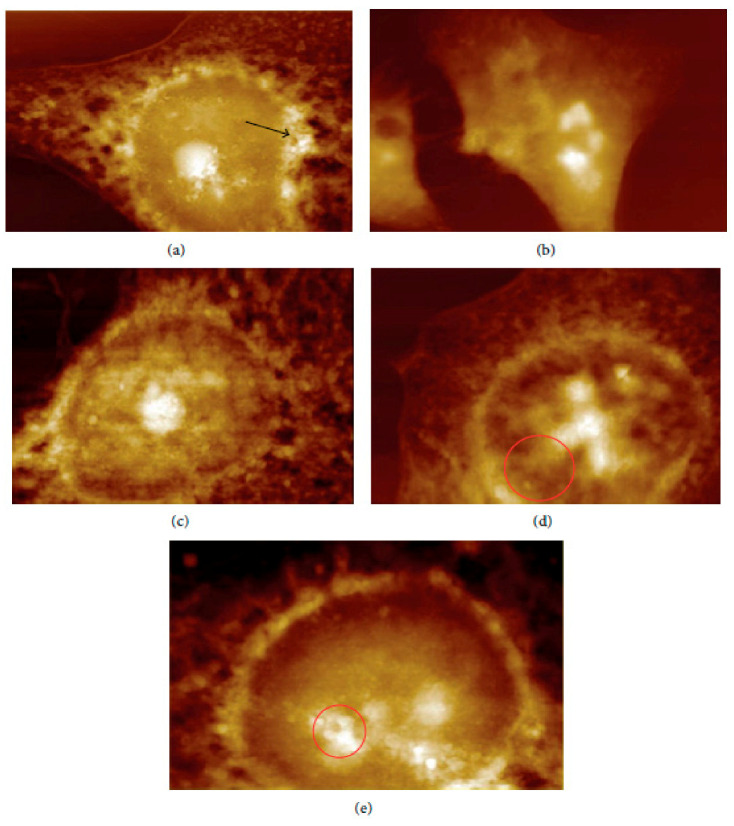
AFM images of 25-nm gold NPs into Hep-2 cells after different exposure times: (**a**) 1, (**b**) 2, (**c**) 4, (**d**) 12, and (**e**) 24 h [65].

In vivo and ex vivo approaches employing an AFM can be used to image NMs in tissues and measure their influence on tissues’ mechanical properties. In ex vivo experiments, animal tissues are isolated and then exposed to an appropriate amount of NMs. In vivo experiments involve the dosing of animals with the NMs followed by tissue isolation, sectioning, and measurement/imaging of the tissues’ quantitative nanomechanical properties of the tissues [64]. For example, Boyoglu and co-workers reported on the cytotoxicity studies of gold NPs into Hep-2 cells. Their studies demonstrated the use of AFM to illustrate cellular localizations of different sizes of gold NPs (e.g., 3 nm, 25 nm) into Hep-2 cells after various exposure times [65]. From their results (Figure 3 and Figure 4), the obtained AFM images show that 3-nm gold NPs entered the nucleus of Hep-2 cells while 25-nm gold NPs accumulated around the Hep-2 cell nucleus as the exposure time increased. According to Boyoglu et al., the access to the nucleus becomes limited as the gold NPs’ size increases because of nuclear membrane restriction, such as the complexity of the nuclear pore (made of different protein components, which favor the nuclear transportation). On the other hand, gold NPs with a small size can quickly enter the nucleus at a fast rate as the exposure time increases and can damage the cellular membrane and kill the Hep-2 cells [65].

#### 4.2.2. Biomimetic 3-D Lung-on-a-Chip

It is made up of two adjoining parallel channels lined with human pulmonary alveolar epithelial cells and human umbilicus endothelial cells, respectively, while the middle channel is packed with 3-D Matrigel. The fluid flow on endothelial cells mimics blood circulation in vivo. Culturing both epithelial cells and endothelial cells on opposite sides of the 3-D extracellular matrix membrane allows cell–extracellular matrix interaction, cell–cell communication, and fluidic stimuli in native alveoli, thus mimicking key structural and physiological characteristics of the alveolar–capillary barrier [66].

#### 4.2.3. Carbon Fiber Microelectrodes (CFMEs)

This amperometry technique is used in single-cell analysis to explore the biophysics of exocytosis, and it is a significant method to understand cellular communication under the influence of nanoparticles [22].

#### 4.2.4. Fluidic-Based Cell-on-Chip (CoC)

Microfluidics offer the ability to introduce many biological conditions in a single device and replicate in vivo situations and dimensions [67]. Therefore, it allows rapid, real-time, and multi-sample analysis, which creates a versatile, non-invasive technique that can produce quantitative information concerning alterations in cellular function upon exposure to different NMs [68,69].

#### 4.2.5. High-Throughput Nanotoxicity Screening

A variety of methods, such as genotoxicity (high-throughput comet assay, γH2AX assay, and high-throughput in vitro micronucleus assay), high content analysis, high-throughput flow cytometry, label-free cellular screening of nanoparticle uptake, impedance-based monitoring, and multiplex analysis of secreted products, can be employed for high-throughput screening and high content analysis for nanoparticle toxicity testing. These methods allow the testing of numerous NPs at different concentrations and on diverse cell types, thus reducing inter-experimental variation while saving cost and time [70,71].

#### 4.2.6. Lateral Flow Immunoassay (LFIA)

It is a paper-based strip biosensor designed to identify specific analytes in a sample. It can be used to measure the concentration of an oxidized guanine base, 8-hydroxy-2′-deoxyguanosine (8-OHdG), formed from ROS-induced oxidative DNA damage, and thus can measure nanotoxicity on the genomic level [72].

#### 4.2.7. Organ-on-Chip

Organs (or tissues) are incorporated on an organ-on-chip platform, which is microfluidic and enables the assessment of nanotoxicity in vitro under extremely dynamic conditions. An organ-on-chip gives more control and can simulate the microenvironment of the tissue under in vivo conditions [53].

#### 4.2.8. Precision-Cut Tissue Slices

It is an ex vivo system compatible with organ samples from diverse species, for example, humans and rodents. Sliced tissues, such as lung slices (200–300 μm), possess different types of cells present in the lungs, including those relevant for inducing immune responses (e.g., mast cells, macrophages, and dendritic cells). Organ-typical cell-cell structures and pulmonary morphology facilitating intercellular communication are sustained till 70 h, thereby reflecting physiological pulmonary histology [73].

In summary, the advanced methods described above also have some limitations, such as their complexity, high operational cost, and that they are time consuming. As a result, an in silico approach has been developed [74,75]. It is one of the novel methods used to evaluate NMs’ toxicity. It takes into consideration the ethical standards, time required, and consistency of results. It is based on the principle of modelling and computational simulation of the results for various physiological properties of NM molecules [74,76]. It produces high-throughput screening data and quantitative structure–activity relationship models to establish a correlation between physicochemical properties and nanotoxicity. In addition, the risk assessment and hazard control of NMs are explored. However, the data produced depend upon reliable experimental toxicity results obtained from in vitro and in vivo studies [6,77,78].

## 5. Toxicity of Carbon, Polymer, and Metallic-Based Nanocomposites and Their Toxicological Side Effects

### 5.1. Carbon Nanotube-Based Nanocomposites

Carbon nanotube-based nanocomposites possess excellent functionalities useful in many applications, such as water treatment, bioengineering, electronics, and renewable energy. Under chronic exposure, in vivo experiments have shown that carbon nanotubes can cause persistent interstitial inflammation and dose-dependent epithelioid granulomatous lesions in the lung. In contrast, in vitro experiments have proved that carbon nanotubes are responsible for stimulating platelets’ aggregation and boosting vascular thrombosis in the rat carotid artery [2]. Moreover, a reduced side effect in cytotoxicity has been reported for carbon nanotubes with a coated or modified surface [79].

### 5.2. Chitosan-Based Nanocomposites

**Figure 5:**
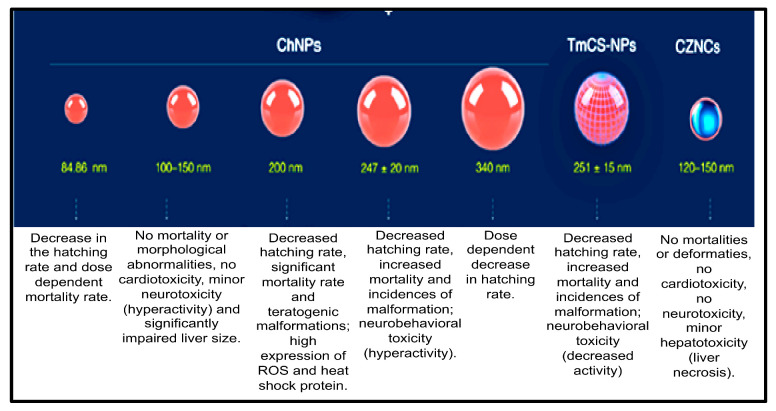
Toxicity evaluation of the different sizes of chitosan-based nanomaterials: summary of the results obtained [80].

Chitosan-based nanocomposites are multifunctional nanomaterials that have been widely applied in combating marine biofouling and also in various fields, such as bioengineering, water purification, medical (for disease detection), pharmaceuticals, as well as drug delivery. Therefore, assessing their toxicity is crucial for their safe use in many applications. Previous studies have investigated the toxicity of chitosan-based nanocomposites using zebrafish embryos as an in vivo model. The nanotoxicity of chitosan-nanoparticles (ChNPs), chitosan-zinc-oxide nanocomposites (ChZNCs), and tween-modified chitosan (TmCh-NPs) has been reported, and a summary of these studies is presented in Figure 5 [80].

Younes and co-workers revealed that ChZNCs may be moderately toxic against the liver, while zebrafish embryos treated with a ChZNCs concentration of 25–200 mg/L did not produce any major effect on the neurological behavior or deformities of the embryos. ChZNCs-treated embryos also showed normal heartbeat functions [81]. The organ-specific toxicity of ChNPs was also investigated in zebrafish embryos by Abou-Saleh et al. [82]. They demonstrated that high concentrations of ChNPs might be toxic against zebrafish embryos as abnormal hyperactivity and significant impairment of the liver size compared to the negative control were observed [82]. In contrast, zebrafish embryos treated with a low concentration of ChNPs displayed normal heart physiology and functional activity. Abou-Saleh and co-workers also used small sizes of ChNPs (100–150 nm), and no toxic effect or teratogenic phenotypes were depicted [82]. The same results were obtained even when a high concentration (200 mg/L) of ChNPs was used. Similarly, Wang et al. also demonstrated that 200 mg/L of ChNPs with a smaller size (84.86 nm) did not induce major mortality in the treated embryos [83].

On the other hand, the study reported by Hu and co-workers revealed that larger ChNPs (340 nm) were less toxic than smaller ChNPs. Particularly, zebrafish embryos treated with a low concentration of 200 nm ChNPs were severely affected, and side effects, such as a bent spine, teratogenic deformities, and pericardial edema, were observed as well as reactive oxygen species formation, leading to increased cell death [84]. Additionally, Yuan et al. (2016) showed that zebrafish embryos treated with tween-80-modified ChNPs develop some toxicity side effects, decreased rate of hatching, increased deformities incidence, and mortality in a dose-dependent manner [85].

From the summary of the results presented in Figure 4, it can be concluded that some biodegradable NMs (e.g., poly-alkyl-cyano-acrylate) can induce the production of undesired products (toxic components) in the organism due to their physicochemical properties. However, most of these biodegradable NMs (chitosan-based nanocomposites, β-cyclodextrin-based nanocomposites) mostly degrade to sugars, amino acids, and fatty acids, which are essential for building of the human body. Hence, these biodegradable NMs are widely considered to be non-toxic [5,86,87].

### 5.3. Cyclodexdrin Nanosponge-Based Composites

Cyclodextrin nanosponges and their hybrid nanocomposites have been proven to exhibit excellent properties, vital for their application in many fields, particularly in drug delivery and water treatment [88]. Previous studies have reported on the nanotoxicity of cyclodextrin nanosponge composites. These studies revealed that cyclodextrin nanosponges synthesized using carbonyl imidazole, hexamethylene diisocyanate, and pyromellitic dianhydride as linking agents are generally non-toxic and safe. For instance, no side effects were noticed when they were administered to mice orally, and these cyclodextrin nanosponges have also shown good blood compatibility [89,90]. Furthermore, some hydroxypropyl derivatives, β-CD, γ-CD, and sulphobutylether-β-CD, seem to be safe and less toxic. Nonetheless, alkylated β-cyclodextrin (β-CD), parents of β-CD, and α-CD are not appropriate for administration because they are renally toxic and toxic through intravenous injection, and can disrupt biological membranes [88,91].

### 5.4. Metallic-Based Nanoparticles

Over the past decades, metallic nanoparticles have also attracted tremendous attention because of their great properties (e.g., high reactivity, small size) and they are useful in many applications, such as medical, industrial, and cosmetic applications. For this reason, the evaluation of their nanotoxicity is crucial to determine the risk associated with their use in many applications [92]. For instance, Matej Skocaj and co-workers conducted short- and long-term toxicity studies of SiO_2_ NPs and TiO_2_ NPs using L6 cells and NPU cells. These authors demonstrated that the sensitivity of L6 cells to NPs was the highest. Transmission electron microscopy analysis confirmed the uptake of NPs into L6 cells but not in NPU cells. Besides that, after 24 h of L6 cell exposure to NPs, there was a reduction in cell viability and increased ROS. Then, the more stable TIO_2_ NPs caused an oxidative damage-associated response after continuous exposure. TiO_2_ NPS also impeded the differentiation of L6 cells [92]. Moreover, Santonastaso et al. were also able to demonstrate the in vitro genotoxic effects of the combined TiO_2_ and CdCl_2_ NPs in human sperm cells [93].

In another study, Konstantin Pikula and co-workers demonstrated the use of sea urchin *Strongylocentrotus intermedius* as a novel approach to assess the nanotoxicity of NMs, such as Au NPs, silicon nanotubes (Si NTs), TiO_2_ NPs, cadmium, and zinc sulfide (CdS and ZnS) nanocrystals. In summary, they observed the following trend in toxicity: Au NPs > Si NTs > CdS > ZnS > TiO_2_ [94].

Additionally, Mariano et al. demonstrated that the green microalgae Chlorella vulgaris can be considered as a model to evaluate the nanotoxicity and bioaccumulation of glucose-capped Ag NPs (AgNPs-G). These authors showed that *C. vulgaris* was capable of taking up AgNPs-G without releasing it back into the medium. The AgNPs-G also did not go through biotransformation (their crystalline nature was maintained) [95].

Further study by Khaled Greish and co-workers showed the effect of Ag NPs on the learning memory, motor function, and social behavior of BALB/C mice. These authors reported that there is damage of these functions in Ag NPs-treated BALB/C mice. Their results also confirmed the evidence that systemic exposure to Ag NPs may result in modification of the cerebral cognition and permits additional consideration of the impact of Ag NPs on human health with respect to their potential neurotoxicity [96].

## 6. Challenges and Limitations

There are some limitations and challenges in interpreting, validating, and correlating cell and tissue toxicity data collected for nanomaterials [97]. The critical challenge in the evaluation of nanotoxicity is the lack of appropriate tools to observe and interrogate nanostructures in complex biological systems directly. For example, suitable interactions of nanostructures with biological systems are lacking, resulting in an unclear understanding of whether the exposure could produce harmful biological responses [97]. Moreover, nanotoxicity’s positive and negative controls need to be identified; the test methods need to be standardized and validated. In addition, harmonization of the data obtained from nanotoxicity experiments must be conducted [3]. Acute versus chronic effects and hazards due to exposure to nanomaterials are also difficult to monitor. Therefore, various measurement techniques must be revised, wisely evaluated (for validity), and used in complex nanomaterial systems [98,99].

The significant limitations are the problems in establishing reasonable in vitro assays (or models) corresponding to in vivo models. Additionally, different particle sizes, surface properties, and poor dispersion of NMs, leading to improper biological distribution and unreliable results, have been reported. Moreover, the interference of carbon-based nanomaterials with toxicity assays and their potential influence on nanotoxicity assessment have also been reported as significant limitations [98]. Previous literature has proved that some NMs can distort the toxicity assay result by adsorbing to the dye’s reagents due to their high surface energy and large surface area to volume ratio. For instance, carbon-based NMs (e.g., single-walled carbon nanotubes, carbon black) have been demonstrated to adsorb MTT-Formazan (a dye used in MTT assays). Specifically, in some immune assays, proinflammatory cytokines can be adsorbed by carbon black, indicating false-negative results and low estimation of its potential toxicity. On the other hand, SWCNTs appear to absorb essential nutrients from cell culture media, leading to indirect cytotoxicity effects, which produce false-positive data and the overestimation of toxicity [45]. Therefore, what should be considered as appropriate biochemical tests for the nanotoxicity evaluation of carbon-based nanomaterials? To answer this question, international standard methods for the evaluation of NM toxicity taking into consideration their physicochemical properties (e.g., size, surface charge, morphology, etc.) need to be approved and researchers are called to follow these approved international standard methods to assess the nanotoxicity of NMs to obtain consistent results in different laboratory settings.

## 7. Conclusions and Recommendations

Nanomaterials and their hybrid nanocomposites possess excellent features beneficial for their use in various fields. It is also important to note that their increased use in many consumer products may lead to some toxicity side effects, which can be investigated using diverse methods. These diverse approaches to assess the nanotoxicity in vitro and in vivo have been discussed. However, the studies reported are not explicit enough to interpret the data. Hence, more research studies on the nanotoxicity of NMs are needed. It is recommended that conventional assays used for toxicity studies are improved by internationally recognized standards of qualification on the toxicity of NMs, then validated by a series of experimental controls for a particular NM variant, restricted to the NM’s concentrations below the levels of interference. This approach can be followed to obtain consistent and trustworthy nanotoxicity data. The validated data can be further processed following the in silico approach, which uses computational simulation and appropriate mathematical models, to predict and better explain the mechanisms and the relationship between the NMs’ physicochemical properties and nanotoxicity.

## Figures and Tables

**Table 1:** In vitro assays for evaluating nanotoxicity.

Parameters	Tests	Description	References
**Proliferation Assay**	DNA Content	Proliferation can be quantified by observing and counting cells in mitosis while agents that hinder or cause mitotic progression are identified. Results are normally represented as a mitotic index (number of cells undergoing mitosis/total number of cells in a given population). Ki-67 nuclear antigen or a compound able to prevent cells in metaphase, such as colchicine, may be used.	[24]
	[3H] thymidine incorporation	This technique requires incubation (24–48 h) with [3H] thymidine. During the S phase, DNA of viable cells pick up [3H] thymidine, thus indicating the number of cells undergoing proliferation.	[35]
	Bromodeoxyuridine (BrdU) incorporation	BrdU has enhanced specificity for cells going through DNA synthesis compared to [3H] thymidine, and a flow cytometry or special antibodies can be used to detect its presence. This assay can be used to investigate the proliferative as well as the antiproliferative effects of nanoparticles.	[24]
	Ki-67 assay	Apart from G0, the nuclear antigen Ki-67 is present in all cell cycle steps and is detected through immunohistochemistry or spectrophotometrically.	[34,36]
**Apoptosis Assay**	Caspase assays	This assay is a luminescence-based test that measures caspase-7 and caspase-3 activities. Upon addition to caspase reagent to cells treated with NP, there is cell lysis followed by substrate cleavage using caspase, and then the luciferase produces a luminescent signal, which is comparable to the available quantity of caspase activity.	[37]
	TUNEL assay (Terminal deoxynucleotidyl transferase dUTP(deoxyuridinetriphoshate) nick end labelling)	It relies on the detection of DNA fragments produced in the last steps of apoptosis; the ends of DNA fragmented by endonucleases are labelled with biotinylated nucleotides conjugated to bromodeoxyuridine (BrdU), which can be identified by making use of a diaminobenzidine chromogen and streptavidin-horseradish peroxidase by fluorescent microscopy, or light microscopy or an immunohistochemical assay.	[38]
	Annexin V	It is commonly utilized to identify apoptotic cells, which bind strongly to phosphatidylserine in a calcium-dependent manner. Phosphatidylserine is usually excluded from the plasma membrane’s extracellular surface but flips from the inner to the outer side upon the onset of apoptosis. The presence of phosphatidylserine on the extracellular surface sustains the membrane’s integrity while signaling for platelet aggregation and macrophage consumption. Calcium-mediated annexin V binds to the exposed phosphatidylserine as a shield from coagulation cascades to hinder rampant blood coagulation from the natural cell cycle. Therefore, the apoptotic cells can be detected using fluorescently labelled Annexin V.	[16,19]
**Genotoxicity Assays**	Ames assay	It is applied to test reverse mutation in *Salmonella typhimurium.* These bacteria have a mutation on the HIS operon and cannot generate the amino acid histidine, which is essential for bacterial replication. Exposing the bacteria to NP enables the bacteria to reverse the HIS operon’s mutation, resulting in histidine generation and colony development, which can be counted. Base-pair substitutions due to reverse mutations at the tryptophan locus can be tested in *Escherichia coli* (WP2*uvr*A).	[24,39]
	Comet Assay	It is used for quantifying and assessing DNA damage in cells. Cells embedded in a thin agarose gel are deposited on a microscope slide, and cellular proteins are obtained from the cells by lysing. The DNA is allowed to uncoil in neutral or alkaline conditions, and then it goes through electrophoresis, enabling the damaged DNA fragments to move away from the nucleus. The degree of fluorescence in the tail length, tail, and head is measured by staining with ethidium bromide or propidium iodide. The extent of DNA liberated from the comet’s head is immediately comparable to the amount of damaged DNA.	[40,41]
	Measurement of oxidized guanine bases	Single base changes within a specific gene can be detected by assaying any one of several oxidized guanine bases, e.g., 7,8-dihydro-oxodeoxyguanine (oxo-dG) and 8-hydroxydeoxyguanosine (8-OHdG). The changes of these bases are frequently a result of oxidative damage and are measured through immunohistochemistry or HPLC.	[42]
	Chromosomal aberration induction	This technique entails evaluating the influence of NPs on the number of cells and changes in chromosomes’ morphological appearance. Since cells are more sensitive during the S-phase, they are treated with NPs at this stage, followed by treatment at planned time intervals with colcemid or colchicine. Trypsinated cells are counted, and chromosomes are prepared and stained with Giemsa stain for microscopic evaluation of aberration (chromatid breaks, chromatid exchange, chromosome breaks, chromosome fragmentation).	[43]

**Table 2:** Organisms used for the evaluation of in vivo nanotoxicity.

Organism	Description	References
***Arabidopsis thaliana***	It is a simple model system because its life cycle is rapid, it can easily be cultivated in a small space, and the information regarding its genome can also be clearly obtained. During the seedling period, *A. thaliana* is exposed to NPs for a given period of time; then, using the microarray technique, genomic analysis of harvested roots and leaves is done.	[52]
***Dapniapulex* and *Daphnia magna***	They are small planktonic crustaceans primarily used for pre-screening since the biological differences between crustaceans and humans are extremely wide. They can be used to evaluate the effect of NP on mortality and reproduction.	[53]
***Caenorhabditis elegans***	After exposing worms to NPs, a customized microfluidic chip is used to measure the level of metallothionein (mtl-2) gene expression, behavior, and worm length/width. Evaluating the physiological responses of *C. elegans* against chronic or acute anti-inflammatory and neurological therapeutics is helpful because the human biochemical pathways correlate well with the metabolic pathways and stress-associated markers of *C. elegans*.	[62]
***Drosophila melanogaster***	Different modes of nanotoxicity like metabolic defects, fecundity, genotoxicity, and oxidative stress can be investigated in *D. melanogaster*.	[63]

## Data Availability

Not applicable.
